# Characterization of *Streptococcus pneumoniae* isolates from Austrian companion animals and horses

**DOI:** 10.1186/s13028-017-0348-2

**Published:** 2017-11-14

**Authors:** Maximilian Ginders, Michael Leschnik, Frank Künzel, Doris Kampner, Claudia Mikula, Georg Steindl, Inga Eichhorn, Andrea T. Feßler, Stefan Schwarz, Joachim Spergser, Igor Loncaric

**Affiliations:** 1 Department of Pathobiology, Institute of Microbiology, University of Veterinary Medicine Vienna, Vienna, Austria; 2 Department for Companion Animals and Horses, University of Veterinary Medicine Vienna, Vienna, Austria; 3 National Reference Laboratory for Pneumococcus, Austrian Agency for Health and Food Safety, Graz, Austria; 4 The Healthcare Company of Styria, Graz, Austria; 5 Centre of Infection Medicine, Department of Veterinary Medicine, Institute of Microbiology and Epizootics, Freie Universität Berlin, Berlin, Germany

**Keywords:** Companion animals, Horses, Multi-drug resistant, *Streptococcus pneumoniae*, Zoonosis

## Abstract

**Background:**

The aim of the present study was to investigate the genetic relatedness and the antimicrobial resistance profiles of a collection of Austrian *Streptococcus pneumoniae* isolates from companion animals and horses. A total of 12 non-repetitive isolates presumptively identified as *S. pneumoniae* were obtained during routinely diagnostic activities between March 2009 and January 2017.

**Results:**

Isolates were confirmed as *S. pneumoniae* by bile solubility and optochin susceptibility testing, matrix-assisted laser desorption-ionization-time of flight (MALDI-TOF) mass spectrometry and sequence analysis of a part *recA* and the 16S rRNA genes. Isolates were further characterized by pneumolysin polymerase chain reaction (PCR) and genotyped by multilocus sequence typing (MLST). Antimicrobial susceptibility testing was performed and resistance genes were detected by specific PCR assays. All isolates were serotyped. Four sequence types (ST) (ST36, ST3546, ST6934 and ST6937) and four serotypes (3, 19A, 19F and 23F) were detected. Two isolates from twelve displayed a multidrug-resistance pheno- and genotype.

**Conclusions:**

This study represents the first comprehensive investigation on characteristics of *S. pneumoniae* isolates recovered from Austrian companion animals and horses. The obtained results indicate that common human sero- (23F) and sequence type (ST36) implicated in causing invasive pneumococcal disease (IPD) may circulate in dogs. Isolates obtained from other examined animals seem to be host-adapted.

## Background

*Streptococcus pneumoniae* is a major human pathogen that colonizes the upper respiratory tract and causes both life-threatening diseases such as pneumonia, sepsis and meningitis but also sinusitis and otitis in both children and adults [1]. *S. pneumoniae* is responsible for community-acquired respiratory tract infections in infants. *S. pneumoniae* infections of pregnant women may be associated with stillbirth and fetal death [2]. Several animal models have been used to study *Pneumococcus*-associated pneumonia, meningoencephalitis and otitis [3, 4]. *S. pneumoniae* is assumed to be a human pathogen only. Nevertheless, there are established mouse and rat models for various *S. pneumoniae*-caused diseases [4]. Zooanthropogenic transmission of other streptococcal species is well documented, in particular for *S. equi* subsp. *zooepidemicus*, *S. canis*, *S. suis*, *S. porcinus* and *S. phocae* [5–9]. Pneumococci can easily exchange DNA in their natural habitat, the human mouth and throat. This environment is populated by several streptococcal species, which form a ‘gene pool’ out of which the pneumococci can recruit resistance genes. Gene transfer and mosaic genes have been intensively reported for *S. pneumoniae* and other streptococci [10]. While comprehensive data on human *S. pneumoniae* infections exists, there is still a lack of information on infections, carriage and zoonotic potential of this particular pathogen in pet and companion animals. Therefore, the aim of the present study was to investigate the genetic relatedness and the antimicrobial resistance pattern of a collection of Austrian *S. pneumoniae* isolates from infections of pet and companion animals as well as horses.

## Methods

A total of 12 non-repetitive isolates, presumptively identified as *S. pneumoniae,* were obtained between 2009 and 2017 during diagnostic examinations at the Institute of Microbiology of the University of Veterinary Medicine, Vienna, Austria. The isolates were identified as *S. pneumoniae* using classical bacteriological methods and originated from guinea pigs (n = 6), horses (n = 3), a dog (n = 1) and pet rats (n = 2). The annual isolation frequency of *S. pneumoniae* was inconstant. All isolates were stored in glycerol stocks at − 80 °C. For the present study, isolates were re-grown on Mueller–Hinton Agar with 5% sheep blood and on Improved II agar [Becton–Dickinson (BD), Heidelberg, Germany]. They were confirmed as *S. pneumonia* by bile solubility [19] and optochin susceptibility testing [11], matrix-assisted laser desorption-ionization–time of flight mass spectrometry (MALDI-TOF MS) (Bruker Daltonik), as well as sequence analyses of a part of the *recA* [12] and 16S rRNA genes [13]. Isolates were further characterized by pneumolysin polymerase chain reaction (PCR) [14] and genotyped by multilocus sequence typing (MLST). MLST was carried out by PCR amplification and sequencing of seven housekeeping genes (*aroE*, *gdh*, *gki*, *recP*, *spi*, *xpt*, *ddl*) as described previously [15]. Allelic profiles and sequence types (ST) were assigned using the MLST database hosted at http://pubmlst.org/spneumoniae/. Antimicrobial susceptibility testing was performed by agar disc diffusion according to the Clinical and Laboratory Standards Institute (CLSI) [16, 17] including oxacillin, tetracycline, doxycycline, erythromycin, clindamycin, chloramphenicol, vancomycin, trimethoprim–sulfamethoxazole, enrofloxacin, marbofloxacin and linezolid (all from Becton–Dickinson, Heidelberg, Germany). With respect to the resistance phenotype, resistance genes were detected by previously described PCRs applying specific primers for *tet*(M), *erm*(B), *mef*(A), *mef*(E), *msr*(E) *cat*_pC194_, *cat*_pC221_ and*cat*_pC223_ [18–21]. The presence of mutations within fragments of the genes encoding either a dihydropteroate synthase (DHPS) that plays a role in conferring resistance to sulfamethoxazole or a dihydrofolate reductase (DHFR) conferring resistance to trimethoprim in *S. pneumoniae* were tested by PCR followed by DNA sequence analysis [22]. Furthermore, PCRs for the detection of two *int*-*Tn* genes encoding the integrases of the conjugative transposons Tn*1545* (conferring resistance to tetracycline, kanamycin and macrolides) and Tn*5252* (chloramphenicol resistance) were performed [21]. Amplicons of *int*-Tn*1545* as well as *int*-Tn*5252* were sequenced. Finally, capsular serotyping was conducted by the Quellung reaction [23] employing sera obtained from Statens Serum Institute (SSI), Copenhagen, Denmark. Typing sera included 12 serum pools for serogrouping and various type-specific antisera. Serotyping was performed according to the manufacturer’s recommendations.

## Results

All tested isolates were bile-soluble, optochin-susceptible and positive for the pneumolysin gene. MALDI-TOF MS identified all strains as *S. pneumoniae*. The *recA* and 16S rDNA sequence analyses showed that all isolates were closely related to the type strain of *S. pneumoniae.* MLST revealed that all guinea pig isolates (n = 6) belonged to sequence type (ST) 6937 (allelic profile 2-5-4-5-27-20-5), all equine isolates (n = 3) were ST6934 (allelic profile 10-9-4-12-287-426-470), the dog and the two rat isolates were ST36 (allelic profile 1-8-4-1-1-4-6) and ST3546 (allelic profile 1-5-41-5-10-28-8), respectively. Susceptibility testing of the isolates showed that all but two isolates were susceptible to all antimicrobial agents tested. Isolates 2946 and 880, both obtained from rats, exhibited resistance to tetracycline, erythromycin, clindamycin, chloramphenicol and trimethoprim-sulfamethoxazole, and were positive for *tet*(M), *erm*(B), *cat*_pC194_, and *int*-*Tn1545* as well as *int*-*Tn5252*. Moreover, they displayed the same mutations in the genes *sulA* and *dfr*, i.e. an insertion of 6 bp within *sulA*, the gene encoding DHPS, resulting in the duplication of amino acids Arg58 and Pro59 as well as mutations within the *dfr* gene that resulted in amino acid exchanges at the position 92 (Asp-92-Arg) and 100 (Ile-100-Leu) of the dihydrofolate reductase. These alterations have previously been described to be associated with sulfamethoxazole and trimethoprim resistance [24]. Serotyping identified all guinea pig isolates as serotype 19F, the three horse isolates as serotype 3, the dog isolate as serotype 23F and the rodent isolates as serotype 19A (Table 1).

**Table 1:** Origin, molecular characterization, antimicrobial resistance and serotypes of the 12 *S. pneumoniae* isolates investigated

Isolate	Year of isolation	Host species	Site of isolation	Symptoms	ST	Resistance phenotype	Resistance genotype	Serotype
649	2009	Guinea pig	Lung	Respiratory	6937	–	–	19F
2704	2009	Horse	Diverticulum tubae auditivae	Respiratory	6934	–	–	3
2902	2009	Horse	Trachea	Respiratory	6934	–	–	3
2946	2009	Rat	Lung	Respiratory	3546	TET, ERY, CLI, CHL, SXT	*tet*(M), *erm*(B), *cat*_PC194_, *dfr*	19A
747	2010	Horse	Trachea	Respiratory (RAO)	6934	–	–	3
1166	2010	Guinea pig	Lung	Central nervous, respiratory	6937	–	–	19F
1409	2010	Guinea pig	Lung	Respiratory	6937	–	–	19F
864	2011	Guinea pig	Lung	Respiratory	6937	–	–	19F
2994	2012	Guinea pig	Pleural punction	Respiratory	6937	–	–	19F
1537	2014	Dog	Cerebrospinal fluid	Central nervous	36	–	–	23F
271	2017	Guinea pig	Ear	Central nervous	6937	–	–	19F
880	2017	Rat	Lung	Respiratory	3546	TET, ERY, CLI, CHL, SXT	*tet*(M), *erm*(B), *cat*_PC194_, *dfr*	19A

## Discussion

A total of 12 non-repetitive *S. pneumoniae* isolates, originated from pet, companion animals and horses were analysed. The comparison of ST/serotype combination with the MLST database (http://pubmlst.org/spneumoniae/) showed the following outcomes. All six guinea pig isolates displayed a ST6937 and serotype 19F combination. Serotype 19F is a common serotype encountered in human isolates; according to the MLST database, which contains both ST and serotypes, it is associated with a large number of different STs and is often linked with invasive pneumococcal disease as well as *S. pneumoniae* carriage. This ST/serotype combination has actually 21 entries in the database, including 19 isolates from guinea pigs in Germany, the Netherlands, France and Peru. Another two isolates were found in a nasal and wound swab from infants in Germany and the Netherlands. It seems to be a typical ST/serotype combination among guinea pigs and this observation might suggest that guinea pigs seem to represent a reservoir for *S. pneumoniae* of this specific sequence type [25] with a still not clearly defined relevance in human hosts. Nevertheless, the close contact between children and their pets could be a risk factor for transmission. Our data also showed that this ST/serotype combination can be pathogenic for guinea pigs (Table 1).

The three equine isolates belong to ST6934 and serotype 3. Serotype 3 is also a serotype commonly found in humans, but in the combination with ST6934, six entries were identified in the MLST database. All but one of the isolates originated from horses in Germany and the UK. Whether this particular serotype plays a role as a zoonotic pathogen remains unclear. Whatmore et al. [9] compared a collection of equine and human pneumococcal isolates of serotype 3 using restriction fragment length polymorphism (RFLP) analysis of housekeeping genes. RFLP revealed that equine pneumococci were indistinguishable from each other but different from human isolates. Thus, the results of the present study are in concordance with previous reports suggested that equine *S. pneumoniae* isolates likely represent a host-restricted subpopulation of pneumococci.

Strain 1537/14 was isolated from a dog suffering from encephalitis, belonging to ST36 and serotype 23F. This ST/serotype combination has apparently not previously been reported from a dog or another animal. In the *S. pneumoniae* MLST database, *S. pneumoniae* ST36/23F is represented by 92 records, which all originate from human hosts. Serotype 23F is one of the most prevalent serotypes involved in IPD [26].

The multidrug-resistant isolates 2946 and 880 originated from rats, kept as pet animals, that suffered from pneumonia. They belonged to ST3546 and serotype 19A. The MLST database revealed five records with this combination. Four of the isolates with this ST/serotype combination were isolated from humans in Germany, Norway and Czech Republic. One isolate was also obtained from a rat in Austria in 2007 with a similar multi-drug resistance profile. Linden et al. [25] also found two pet rats with the same ST/serotype combination. Rats are often kept by teenagers in close physical contact and suffer from pneumonia resistant to therapy [27]. It is recommended to examine those pets for the carriage of bacterial pathogens and—if positive—also by antimicrobial susceptibility testing more frequently than it is currently done. Serotype 19A is a common serotype found in humans suffering from IPD [28]. The epidemiology of this serotype is constantly changing. Possible reasons for this are the introduction of pneumococcal conjugate vaccination, increased use of antibiotics, import of multidrug-resistant isolates and increased reporting. The prevalence of serotype 19A, for example in Germany, has increased significantly between 2007 and 2011 [29].

Serogroup 19 (12.8%) as well as serogroup 3 (8.6%) are some of the most prevalent serogroups in humans in Europe. Among the most commonly reported serogroups, dual non-susceptibility to penicillin and macrolides was mainly observed in serogroup 19. Serotypes 19F and 23F, which were detected during the present study from guinea pigs and a dog, are known as common ‘classic’ resistant serotypes [30].

The isolates found in dogs and eventually in rats seem to be associated with human pneumococcal isolates, but further research is needed to improve the knowledge of the zoonotic potential of these bacteria. All companion animals considered in this study, suffered from severe respiratory or central nervous symptoms (Table 1). This strongly suggests that pneumococci are able to cause serious diseases in different animal species.

We recognize that the results of our study have limitations, because the examined collection comprises only twelve isolates. However, the frequency of *S. pneumoniae* isolation is in approximate concordance with previously described by van der Linden [25], who identified 41 strains during 22 years from pets and zoo animals.

## Conclusions

Due to the close contact between companion animals and humans, isolation of human-associated clones in diseased companion animals deserves special consideration, particularly if those isolates display multidrug resistant pheno- and genotypes. The results of the present study confirm that a closer collaboration between human medicine and veterinary medicine is needed.

## Abbreviations

CLSI: clinical and laboratory standards institute
DHFR: dihydrofolate reductase
DHPS: dihydropteroate synthase
IPD: invasive pneumococcal disease
MALDI-TOF: matrix-assisted laser desorption-ionization-time of flight
MLST: multilocus sequence typing
PCR: polymerase chain reaction
RFLP: restriction fragment length polymorphism
ST: sequence type
